# High-throughput quantitation method for amodiaquine and desethylamodiaquine in plasma using supported liquid extraction technology

**DOI:** 10.1016/j.jchromb.2021.122887

**Published:** 2021-08-01

**Authors:** Karnrawee Kaewkhao, Joel Tarning, Daniel Blessborn

**Affiliations:** a Mahidol Oxford Tropical Medicine Research Unit, Faculty of Tropical Medicine, Mahidol University, Bangkok, Thailand; b Centre for Tropical Medicine & Global Health, Nuffield Department of Clinical Medicine, University of Oxford, Oxford, UK

**Keywords:** Amodiaquine, Plasma, LC-MS/MS, Method validation

## Abstract

Amodiaquine is a drug used for treatment of malaria and is often used in combination with artesunate in areas where malaria parasites are still susceptible to amodiaquine. Liquid chromatography tandem-mass spectrometry was used to quantify amodiaquine and its active metabolite, desethylamodiaquine, in plasma samples. A low sample volume of 100 µl, and high-throughput extraction technique using a supported liquid extraction (SLE^+^) technique on an automated liquid handler platform for faster sample processing are some of the advantages of this method. Separation of amodiaquine from desethylamodiaquine was achieved using a reversed phase Zorbax SB-CN 50 mm × 4.6 mm, I.D. 3.5 µm column with acetonitrile and 20 mM ammonium formate with 1% formic acid pH ~ 2.6 (15–85, v/v) as mobile phase. The absolute recoveries of amodiaquine and desethylamodiaquine were 66% to 76%, and their isotope label internal standard were in the range of 73% to 85%. Validation results of the developed method demonstrated intra-batch and inter-batch precisions within the acceptance criteria range of ± 15.0%. There were no matrix or carry-over effects observed. The lower limit of quantification was 1.08 ng/ml for amodiaquine and 1.41 ng/ml for desethylamodiaquine. The method showed robust and accurate performance with high sensitivity. Thus, the validated method was successfully implemented and applied in the evaluation of a clinical trial where participants received artemether–lumefantrine plus amodiaquine twice daily for three days (amodiaquine dose of 10 mg base/kg/day).

## Highlights

• Supported liquid extraction is easy to use as visual phase separation isn't needed.
• Automated sample preparation allows fast sample process and subsequent analysis.
• Automated liquid handling coupled with LC-MS/MS as high-throughput method.
• Washout gradient in LC method removes strongly retaining compounds from the column.
• Sensitive method of 1.08/1.41 ng/ml for amodiaquine/desethylamodiaquine.

## 1 Introduction

Amodiaquine is an anti-malarial drug with a relatively short terminal elimination half-life (t_1/2_ ~ 27 hrs [1]) that is metabolized *in-vivo* to its slowly eliminated active metabolite, desethylamodiaquine (t_1/2_ ~ 11 days [1]), which confers the majority of the anti-malarial effect [1], [2]. Amodiaquine in combination with artesunate is a recommended first-line drug treatment for uncomplicated *Plasmodium falciparum* malaria [3]. Amodiaquine also has high efficacy against chloroquine resistant *P. vivax*, *P. ovale*, *P. malariae* and *P. knowlesi* parasites [3], [4]. However, amodiaquine is structurally similar to chloroquine and cross resistance has developed in some areas and needs to be monitored [5]. The slow elimination of its metabolite, desethylamodiaquine, requires a highly sensitive assay to measure the sustained low concentrations in the elimination phase [6], [7], [8], [9], [10], [11], [12]. Plasma has been used traditionally as a sample matrix because it often allows for simple extraction techniques to be used to remove endogenous components from the sample, compared to other sample matrices such as whole blood. A high-throughput method for sample clean-up and analysis is desirable for large pharmacokinetic studies with large batch of samples to process.

Previously published methods for separation and quantification of amodiaquine have used liquid chromatography (LC) in combination with ultraviolet (UV), fluorescence, electrochemical, or mass spectrometry (MS) detection (Table 1). However, published LC-UV methods require the same or greater sample volume to obtain sufficient sensitivity, and long analysis runtimes. LC-MS has become the gold standard in clinical pharmacology application, and offers higher sensitivity and selectivity. However, previously published LC-MS methods also present a few limitations similar to those for conventional LC-UV which used non-selective protein precipitation for sample extraction that still leave phospholipids and other residues in the sample. Some methods use more efficient sample extraction technique (e.g., SPE) and still maintained very good sensitivity. However, considering how time consuming SPE techniques can be, consisting of several labour-intensive steps and the total cost of SPE [13], [14] this might have an impact on time and cost for larger studies.

**Table 1:** Published quantitation methods of amodiaquine and desethylamodiaquine.

Drug /Metabolite	Matrix/Sample volume (µl)	Extraction method	Column Type	Elution of solvent system	Detection method	Extraction Recovery (%)	Injection volume (µl)	Analysis run time (min)	LLOQ	Reference
Amodiaquine/desethylamodiaquine	Plasma/100	Supported Liquid Extraction plates (SLE^+^)	Zorbax SB-CN 50 mm × 4.6 mm, 3.5 µm	Isocratic	MS/MS	66–70/69–74	2	6.5	1.08/1.41 ng/ml	Developed method
Amodiaquine/desethylamodiaquine	Plasma/250	SPE	Hypersil Gold 100 mm × 4.6 mm, 5 µm	Isocratic	MS/MS	85/86	5	*3.5	0.250/1.50 ng/ml	[14]
Amodiaquine/desethylamodiaquine	Plasma/200	Protein precipitation	Atlantis® dC18 50 mm × 2.1 mm, 3 µm	Gradient	MS/MS	91–97/94–98	10	21	0.3/0.3 ng/ml	[21]
Amodiaquine	Plasma/1000	LLE	µBondapak Rad-Pak Phenyl 100 mm X 8 mm, 10 µm	Isocratic	UV	60	50	*8	5 ng/ml	[22], [23]
Amodiaquine/desethylamodiaquine	Plasma/500	LLE	LiChrospher Si (E. Merck)-based normal-phase 250 mm × 4 mm, 5 µm	Isocratic	UV	83/75	100	*24	5/5 ng/ml	[24]
Amodiaquine/desethylamodiaquine	Plasma/200	Protein precipitation	Agilent Zorbax C18 150 mm × 4.6 mm, 5 µm	Isocratic	DAD	71–93/63–78	20	*8	100/100 ng/ml	[9]
Amodiaquine/desethylamodiaquine	Plasma/100	LLE	Zorbax SB C18, 75 mm × 4.6 mm, 3.5 µm	Isocratic	UV	70–97/75–81	100	*12	35.6 /32.8 ng/ml	[25]
Amodiaquine	Plasma/100	SPE	Kromasil C8 150 mm × 4.6 mm, 5 µm	Isocratic	MS/MS	89	15	4	0.30 ng/ml	[13]

*Approximate total runtime based on figures and chromatographic conditions.

Here, we present a fast LC separation linked with a sensitive tandem mass spectrometry quantification method (LC-MS/MS), with a simple and efficient low sample volume preparation process, for amodiaquine and desethylamodiaquine determination in clinical plasma samples. Low plasma sample volume is an advantage when collecting clinical field samples due to practical and/or ethical constraints (i.e. resource-limited settings, interrupted/incomplete sampling procedures due to patient care, pediatric or pregnant patients).

## 2 Materials & methods

### 2.1 Chemicals and reagents

All reference standards, amodiaquine (purity 100%), desethylamodiaquine (purity 99.4%) and the stable isotope-labeled internal standards (amodiaquine-D10 and desethylamodiaquine-D5, purity > 99%) were acquired from AlsaChim (Illkirch, France). MS grade water, acetonitrile and methanol, and HPLC grade ethyl acetate were obtained from JT Baker (Phillipsburg, USA). MS grade formic acid (98–100%) and ammonium formate were obtained from Fluka (Sigma-Aldrich, MO, USA). Analysis grade ammonium hydroxide 0.5 M was prepared from ammonia solution 25% (Merck, Darmstadt, Germany). Blank plasma with citrate phosphate dextrose (CPD) were obtained from the Thai Red Cross, Bangkok, Thailand. Blank plasma with other anticoagulants (Na-heparin, Li-heparin, fluoride-heparin and fluoride-oxalate) were obtained from healthy volunteers at the Faculty of Tropical Medicine, Mahidol University, Thailand (ethical approval: MUTM 2017–014-01).

### 2.2 Standards and working solutions

Stock solutions (1 mg/ml) of amodiaquine, desethylamodiaquine, amodiaquine-D10 and desethylamodiaquine-D5) were dissolved in water-acetonitrile (50–50, v/v) containing 1% formic acid and stored at −80 °C. Working solutions were diluted in acetonitrile–water (50–50, v/v) and used for spiking of plasma. All solutions were allowed to equilibrate to room temperature before use.

### 2.3 Calibration standards and quality control (QC) samples

The concentrations of the amodiaquine/desethylamodiaquine calibration range were 1.08–263/1.41–610 ng/ml in plasma. Three QC levels of amodiaquine/desethylamodiaquine were prepared at 3.19, 30.7, 226/4.64, 56.4, 524 ng/ml. The lowest and highest concentration in the calibration range represent the lower limit of quantification (LLOQ) and upper limit of quantification (ULOQ), respectively. Over-curve samples were prepared at about 3 × ULOQ and diluted ten-times with blank plasma before analysed as a test of dilution integrity.

### 2.4 Sample preparation

An automated liquid handler platform (Freedom Evo 200, TECAN, Mannedorf, Switzerland) was used for the sample preparation process. Plasma 100 µl was aliquoted into a 96-wellplate and extracted using 350 µl ammonium hydroxide 0.5 M containing stable isotope-labelled internal standards (8.08 ng/ml of D10-amodiaquine and D5- desethylamodiaquine). The pre-treated plasma samples were mixed on a Mixmate (Eppendorf, Hamburg, Germany) (1000 rpm, 2 min) and centrifuged (1100 × g, 2 min). Then, 200 µl of extracted samples were loaded onto supported liquid extraction SLE^+^ 96-well plate (ISOLUTE SLE^+^, 820–0200-P01, IST, Biotage, Uppsala, Sweden) and vacuum (3–4 in. Hg) was applied for 30 s until the wells became dry. Five minutes were allowed for the liquid to fully absorb to the sorbent before elution of the bound sample molecules with 800 µl of ethyl acetate (gravity flow, no vacuum). Thereafter, vacuum (1 in. Hg) was applied for 1 min to complete the elution. The eluated samples were evaporated under nitrogen gas at 70 °C (TurboVap® 96, Biotage) and the dried samples were reconstituted in 800 µl of mobile phase (acetonitrile-ammonium formate 20 mM with 1% formic acid (15–85, v/v).

### 2.5 LC-MS/MS

The LC system was an Agilent 1260 infinity system consisting of a binary LC pump, a vacuum degasser, a temperature-controlled micro-well plate autosampler set at 4 °C and a temperature-controlled column compartment set at 40 °C (Agilent technologies, CA, USA). Data acquisition and processing were performed using Analyst 1.6.2 (Sciex, MA, USA). The analytes were separated on a Zorbax SB-CN 50 mm × 4.6 mm, I.D. 3.5 µm (Agilent Technologies), with a pre-column CN AJO-4305 4 mm × 3 mm, I.D. 3.5 µm (Phenomenex, Torrance, California, USA), at a flow rate of 700 µl/min. The mobile phase consisted of (A) acetonitrile-ammonium formate 20 mM with 1% formic acid pH ~ 2.6 (15–85, v/v) and (B) methanol–acetonitrile (75–25, v/v). The mobile phase gradient was A: 0–2 min, B: 2.2–3.7 min and A: 3.9–6.5 min (with 0.2 min linear gradient switch), resulting in a total runtime of 6.5 min per sample. The injection volume was 2 µl.

An API 5000 triple quadrupole mass spectrometer (Sciex, MA, USA) with a TurboV ionization source interface, operating in the positive ion mode, was used for the MS/MS analysis. Ion spray voltage was set to 5500 V, with a drying temperature at 650 °C. The curtain gas (CUR) was 25 psi and the nebulizer (GS1) and auxiliary (GS2) gases 60 psi. All used collision energy of 29 V.

Quantification was performed using selected reaction monitoring (SRM) for the transitions *m*/*z* 356.15 → 283.2 and 366.15 → 283.15 for amodiaquine, and D10-amodiaquine, and 328.1 → 283.15 and 333.15 → 283.15 for desethylamodiaquine and D5-desethylamodiaquine, respectively.

### 2.6 Method validation

Method validation was performed according to the US Food and Drug Administration (FDA), 2018 [15] and European Medicines Agency (EMA), 2012 guidelines [16]. Four independent validation runs were performed for the calibration curve, accuracy and precision tests. Weighted (1/x and 1/x^2^) and non-weighted linear regression models were evaluated for the calibration curve. The best performing model was chosen based on the accuracy of back-calculated concentrations of the calibration curves and quality control (QC) samples from four runs [17]. Accuracy and precision were calculated as mean relative error (%) and coefficient of variation (%CV), respectively. A single factor ANOVA was used for precision calculations (intra-batch, inter-batch and total-assay variability).

Selectivity and matrix effects evaluation were performed using plasma from six different donors, different anticoagulants (Na-heparin, Li-heparin, fluoride-heparin, CPD, and fluoride-oxalate), and co-administered antimalarial drugs. All these tests were performed through post-column infusion and then as a regular analysis run to confirm that there was no signal that potentially could interfere with the drug identification and measurement. Post-column infusion was a mix of amodiaquine, desethylamodiaquine and their stable isotope-labelled internal standards solution (20 ng/ml) and was used to evaluate signs of signal enhancement or suppression. Co-administered antimalarial drugs were injected individually at a drug concentration of 30 ng/ml (piperaquine, pyronaridine, artesunate, primaquine, carboxyprimaquine, chloroquine, desethylchloroquine) while performing post-column infusion.

Process efficiency and absolute recovery were determined by five replicates of extracted QC sample compared to neat solution and post extraction spiked blank plasma samples.

Each calibration curve was constructed using duplicate samples at each concentration. Intra-day accuracy and precision were evaluated by analysis of five replicates of LLOQ, ULOQ, over-curve and three QC levels. The inter-day accuracy and precision were assessed by analysing four plasma precision and accuracy batches over four days.

Carryover effects were investigated by injecting five ULOQ samples followed by three blank samples. A signal higher than 20% of LLOQ in the injected blank samples would indicate carryover.

Stability of amodiaquine and desethylamodiaquine in plasma was investigated by exposing the samples to five freeze (-80 °C) and thaw (22 °C) cycles. Short term stability at ambient temperature (22 °C) and in refrigerator temperature (4 °C) was investigated at 4 hrs, 24 hrs and 48 hrs. Long term stability of spiked samples in storage condition (-80 °C) was also evaluated. Other stability tests; stability of extracted samples in extraction solution at 4 °C for 24 hrs, stability of evaporated samples at 4 °C and −80 °C, and LC autosampler at 4 °C, were investigated during the validation process.

### 2.7 Clinical applicability

The validated plasma method was applied to a clinical study to quantify amodiaquine and desethylamodiaquine concentrations from study samples.

## 3 Result and discussion

### 3.1 Optimization of LC-MS/MS and sample extraction

A number of reversed phase columns (C18 and CN) were evaluated along with mobile phase optimization. Amodiaquine and desethylamodiaquine were separated adequately on a Zorbax SB-CN 50 mm × 4.6 mm I.D. 3.5 µm column. The optimized LC method had a total run time of 6.5 min, including a washout gradient with methanol: acetonitrile (75:25, v/v). A relatively slow washout gradient was used to flush out any strongly retained compounds that might otherwise accumulate on the column and reduce the column performance over time, or co-elute with the analytes potentially causing signal interference [18], [19]. The selected reversed phase column and optimized LC method had good retention and separation of amodiaquine and desethylamodiaquine. It also provided a shorter analysis run time and better sensitivity than many previously published methods.

**Fig. 1:**
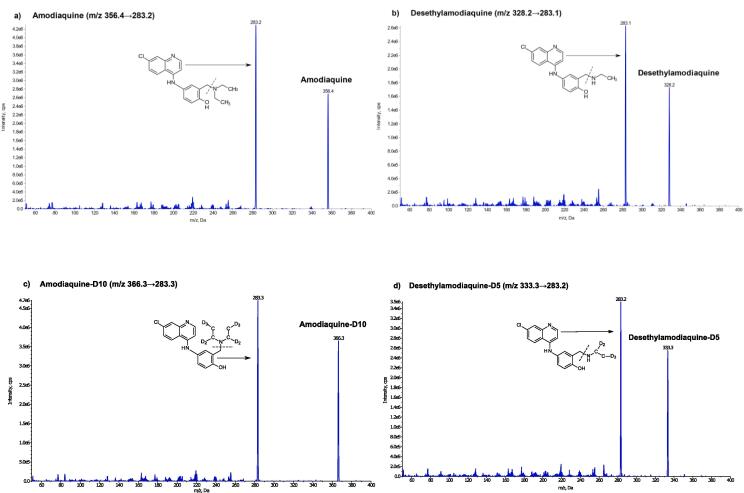
Cumulative collision energy scan and the product ion formed of a) amodiaquine (*m*/*z* 356.4), b) desethylamodiaquine (*m*/*z* 328.2), c) amodiaquine-D10 (*m*/*z* 366.3) and d) desethylamodiaquine-D5 (*m*/*z* 333.3) [20]. The dash line indicates the lost fragment during fragmentation and atom D represent Deuterium.

Optimization of mass parameters was made by manual compound tuning in the positive electrospray ionization (ESI) mode. The final selection of the product ion formed for quantification was based on the most abundant transition signals, selectivity, and sensitivity (as measured by signal-to-noise ratio). However, only one major product ion was produced in the collision cell (Fig. 1). Thus, quantification was performed using SRM transitions of *m*/*z* 356.4 → 283.2 and 366.3 → 283.3 for amodiaquine and amodiaquine-D10, respectively, and 328.2 → 283.1 and 333.3 → 283.2 for desethylamodiaquine and desethylamodiaquine-D5, respectively. The same product ion (*m*/*z* 283) was produced from all amodiaquine analytes and internal standards due to similarities in the molecular structure and the loss of the amino side chain on the phenol ring [20]. This did not have any negative impact on the analyte detection or quantification. The developed detection method resulted in an unbiased robust method with high sensitivity. Previous publications of amodiaquine and desethylamodiaquine methods have shown low sensitivity or used large sample volumes to achieve adequate sensitivity (Table 1).

Previously published methods (Table 1) have used liquid–liquid extraction (LLE), solid-phase extraction (SPE) and protein precipitation. These techniques can be time-consuming and generate large volumes of organic solvent waste or leave hydrophobic contaminants remaining in the final sample (e.g. residual phospholipids) from either protein precipitation or LLE. Consequently, residues can clog the tubing or adsorb to the stationary phase of the reversed phase column. Even though SPE can be more efficient in removing the remaining residuals than protein precipitation or LLE, it is usually more time-consuming since it contains four main steps (conditioning, sample loading, washing and sample elution) in the clean-up process. The technique of LLE has one drawback in that it can sometimes be difficult to see where the two non-miscible liquids are separated and to collect only the desired solvent phase. However, sample clean-up is highly recommended for all analytical techniques to prevent carryover, contamination and unexpected problems in routine analysis of clinical studies. In the sample extraction technique presented here, a supported liquid extraction (SLE^+^) technique for plasma was used. This extraction technique removes proteins and phospholipids from biological samples very quickly. Visual phase separation is not required as the aqueous phase will absorb onto the sorbent bed and analytes can be extracted and eluted by passing through a suitable organic solvent. Therefore, this extraction method is ideal for implementation in the routine drug analysis of large clinical pharmacokinetic trials. Finally, excellent validation results were achieved using this sample extraction technique in combination with the optimized separation and quantitation of amodiaquine and desethylamodiaquine.

### 3.2 Selectivity, sensitivity and matrix effects

**Fig. 2:**
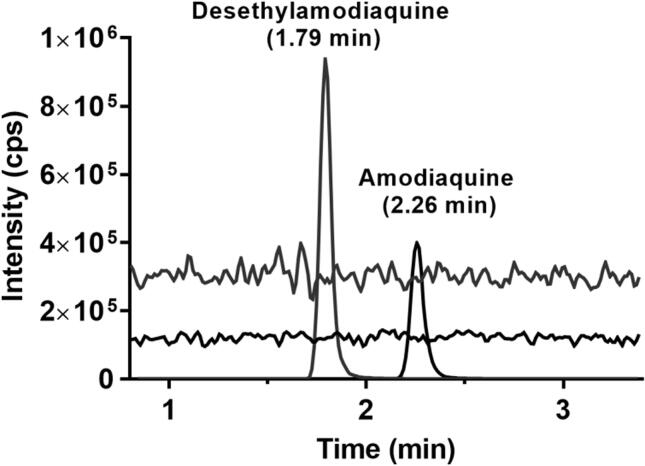
Chromatographic response of amodiaquine and desethylamodiaquine at ULOQ overlaid with the signal of a blank extracted EDTA plasma sample injected during post-column infusion. Infusion was performed at a flow rate of 10 µl/min using a solution containing amodiaquine (20 ng/ml) and desethylamodiaquine (20 ng/ml), showing no interference at the retention times of the analytes.

**Fig. 3:**
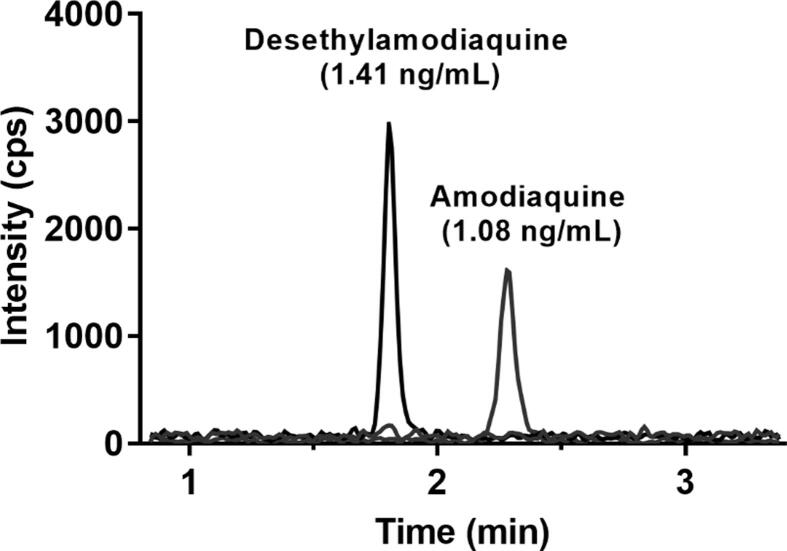
Extracted ion chromatogram of an analysed plasma sample containing LLOQ concentrations of amodiaquine (1.08 ng/ml) and desethylamodiaquine (1.41 ng/ml), overlaid with blank samples.

None of the six blank donor sources or the different anticoagulants of plasma produced a signal contributing >20% of the signal from a standard sample at LLOQ, or >5% of an average isotope label internal standard peak. Therefore, this method was highly selective with minor risk of interference from different individuals. All blank sources extracted post spiked and compared with a neat solution of same nominal concentration were also free from any signs of ion suppression or enhancement for amodiaquine, desethylamodiaquine and their stable isotope-labelled internal standards, resulting in normalised matrix effects close to 1 (Table 2). Post-column infusion while injecting extracted blank plasma sample from blank sources (six different donors and different anticoagulants) also confirmed that there was no visual suppression or enhancement at the retention time of amodiaquine, desethylamodiaquine and their stable isotope-labelled internal standards (Fig. 2, Table 2). Moreover, injection of other commonly used antimalarial drugs (30 ng/ml each of piperaquine, pyronaridine, artesunate, primaquine, carboxyprimaquine, chloroquine, and desethylchloroquine) during post-column infusion did not produce any interfering peaks. The possible co-administered antimalarial drugs were selected as some were of similar chemical structure or used as the first-line treatment in ACTs program as artesunate partner drugs. Therefore, blood samples drawn from malaria patient may have these drugs already present, which can cause further interference to amodiaquine and its metabolite quantification. Overall, there was no matrix interference that would have an impact on the quantification of amodiaquine or desethylamodiaquine. Sensitivity of the method was evaluated as a signal-to-noise response (>10:1) of extracted plasma samples at LLOQ, resulting in clearly visible peaks of amodiaquine and desethylamodiaquine while achieving precision and accuracy within the validation guideline criteria (Fig. 3) [15]. The signal-to-noise response of the analytes at LLOQ were substantially above the threshold of 10:1, but this sensitivity was judged sufficient since it is still more than a 200-fold lower compared to clinical peak levels of desethylamodiaquine, which confers the majority of the antimalarial effect [2].

**Table 2:** Absolute recovery, process efficiency and matrix effect of amodiaquine, desethylamodiaquine and their stable isotope-labelled (SIL) internal standard in human EDTA plasma sample.

Drug	Concentration (ng/ml)	Absolute recovery (%)	CV (%)	Process efficiency (%)	Matrix factor	Normalized matrix factor (drug/IS)	CV (%)
Amodiaquine	QC1: 3.19	69.5	8.73	70.1	1.01	1.01	3.27
	QC3: 226	65.8	4.90	66.0	1.00	1.01	4.11
SIL Amodiaquine-D10	QC1: 8.08	79.3	6.10	79.0	0.996	–	–
	QC3: 8.08	74.3	9.27	74.0	0.996	–	–
Desethylamodiaquine	QC1: 4.64	76.4	5.97	73.6	0.963	0.992	6.48
	QC3: 524	69.7	4.20	69.4	0.996	0.953	6.45
SIL Desethylamodiaquine-D5	QC1: 8.08	84.8	2.75	82.4	0.972	–	–
	QC3: 8.08	72.7	3.55	76.1	1.05	–	–

Five replicates of each QC1 and QC3 level were quantified.

### 3.3 Process efficiency and recovery

The process efficiency (extracted/neat solutions) and absolute recovery (extracted/post extraction spiked) was in the range of 66–85% for plasma at low and high QC levels tested for amodiaquine, desethylamodiaquine and their stable isotope-labelled internal standards (Table 2). The extraction recovery result seems to be lower than some of the published methods (Table 1), however, the recoveries can be different depending on the extraction technique utilized. This method used high-throughput extraction technique, SLE^+^, that can improve chromatographic performance by it’s efficiency to remove phospholipid residues from plasma sample. Therefore, the recovery may be equal or lower than some of the other methods, but it is reproducible and that is crucial for routine analysis. Moreover, the precision (% CV) of recovery (Table 2) was well below the 15% validation limit and this method achieved the desired sensitivity.

### 3.4 Linearity, accuracy and precision

Linearity, accuracy and precision were analysed over four days. Amodiaquine and desethylamodiaquine in plasma quantified using linear regression with 1/x^2^ weighting, resulted in the best prediction of back calculated values for calibration curves and QC samples [26]. Each calibration curve showed a high correlation coefficient (r > 0.997). The intra-day and inter-day precision and accuracy for amodiaquine and desethylamodiaquine gave accuracy values within the range of 93.7–104%. The inter-day and intra-day precision (%CV) varied from 1.64% to 12.6% (Table 3). Both accuracy and precision were well within the allowed regulatory criteria of less than 15% deviation (20% at LLOQ).

**Table 3:** Accuracy and precision for amodiaquine and desethylamodiaquine extracted from human EDTA plasma sample.

		Amodiaquine					Desethylamodiaquine				
		Nominal Concentration(ng/ml)	Measured concentration(ng/ml)	Accuracy (%)	Precision (%)		Nominal Concentration(ng/ml)	Measured concentration(ng/ml)	Accuracy (%)	Precision (%)	
					Inter-assay CV	Intra-assayCV				Inter-assayCV	Intra-assayCV
	LLOQ	1.08	1.09	101	9.47	9.11	1.41	1.47	104	9.45	8.65
	QC1	3.19	3.08	96.5	4.09	5.40	4.64	4.57	98.3	4.41	4.84
	QC2	30.7	30.4	99.2	5.00	3.13	56.4	54.4	96.4	5.10	3.80
	QC3	226	229	101	1.64	2.91	524	491	93.7	12.0	4.48
	ULOQ	263	264	101	4.66	3.73	610	595	97.7	12.6	3.61
	Over curve	828	840	101	2.98	3.35	1932	1874	97.0	5.72	4.60

Five replicates of samples were analysed during four days. Over curve, that is, sample dilution integrity test (1:10 dilutions).

### 3.5 Carry over and stability

Neither amodiaquine, desethylamodiaquine, amodiaquine-D10 nor desethylamodiaquine-D5, produced any detectable carry-over signal after injection of five replicates of ULOQ in the validation tests.

Amodiaquine and desethylamodiaquine in plasma were stable during all stability tests i.e. stability during five freeze/thaw cycles, short term stability at 22 °C and 4 °C (4 hrs, 24 hrs and 48 hrs), extracted samples stability (stored extracted sample at 4 °C for 24 hrs). Evaporated extracted plasma sample stability showed that amodiaquine and desethylamodiaquine were stable for at least 48 hrs at 4 °C and at least 120 hrs at −80 °C when stored as a dried sample. The LC autosampler stability test at 4 °C, showed that amodiaquine and desethylamodiaquine were stable for at least 74 hrs in the autosampler. The long-term storage stability evaluation demonstrated that amodiaquine and desethylamodiaquine were stable for at least 1.6 years at −80 °C (Table S1).

### 3.6 Clinical applicability of validated method

**Fig. 4:**
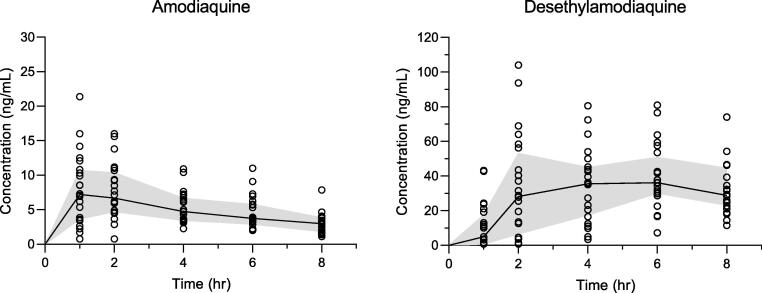
Plasma concentration–time profiles of amodiaquine and desethylamodiaquine after oral administration of amodiaquine twice daily for three days (10 mg base/kg/day) in patients with acute uncomplicated *P. falciparum* malaria [27].

The validated method was implemented and applied in the evaluation of a clinical trial investigating triple artemisinin-based combination therapies (TACTs), in which amodiaquine was administered to patients [27]. Study participants received artemether–lumefantrine plus amodiaquine twice daily for three days (amodiaquine dose of 10 mg base/kg/day). The resulting pharmacokinetic plasma concentration–time profiles are presented in Fig. 4. Amodiaquine is quickly and extensively metabolized in-vivo to desethylamodiaquine, leading to substantially higher mean plasma concentration of desethylamodiaquine [6], [7], [8], [9], [10], [11], [12]. Therefore, the calibration curve of desethylamodiaquine had a wider range with a higher ULOQ than that of amodiaquine to cover the extended range of therapeutic concentrations. Also, the middle QC was positioned lower in the calibration range to where most of the clinical study sample concentrations were expected to be, to give a better estimate of the precision and accuracy of analysed study samples. Repeated analysis of patient samples (10% of total samples), known as incurred sample reanalysis (ISR) showed good results with values deviating less than 20% of the original values. Thus, the developed method proved reliable with robust performance for the analysis of amodiaquine and desethylamodiaquine in clinical trial plasma samples. [15], [28].

## 4 Conclusion

A robust and accurate LC–MS/MS method was developed and validated for the quantification of amodiaquine and its active metabolite, desethylamodiaquine, in plasma samples. The LC method used a washout gradient with methanol: acetonitrile (75:25, v/v) to remove strongly retaining components that otherwise could potentially co-elute with the analytes in subsequent injections and cause signal interference. Therefore, having a washout gradient is an advantage when analyzing large amount of plasma samples. The developed method allowed for accurate and reliable quantification of amodiaquine/desethylamodiaquine down to 1.08/1.41 ng/ml in patient samples, despite using a low sample volume of only 100 µl plasma. The use of supported liquid extraction plates implemented for automated plasma sample preparation on a liquid handler platform and the short chromatographic analysis time of 6.5 min makes this method suitable for large batches of clinical patient samples. This high-throughput method was proved to be reliable and reproducible when implemented in routine clinical trial sample analysis.

## Declaration of Competing Interest

The authors declare that they have no known competing financial interests or personal relationships that could have appeared to influence the work reported in this paper.
