# Supplementary material for: High-throughput quantitation method for amodiaquine and desethylamodiaquine in plasma using supported liquid extraction technology
Source: J Chromatogr B Analyt Technol Biomed Life Sci. 2021 Aug 1;1179:122887. doi: 10.1016/j.jchromb.2021.122887 (PMC8417464; doi:10.1016/j.jchromb.2021.122887)
Supplement: Supplementary data 1 [file mmc1.docx]

**Table S1.** Stability tests of amodiaquine and desethylamodiaquine in human EDTA plasma sample (n=3).

| Drug | Stability Test | | Concentration (ng/ml) | Average ± SD (ng/ml) | Accuracy (%) | Precision (%) |
| --- | --- | --- | --- | --- | --- | --- |
| Amodiaquine | Freeze/ Thaw | Cycle I | QC1: 3.19 | 2.99 ± 0.14 | 93.8 | 4.75 |
|  |  |  | QC3: 226 | 213 ± 9.54 | 94.2 | 4.48 |
|  |  | Cycle III | QC1: 3.19 | 2.84 ± 0.13 | 89.1 | 4.48 |
|  |  |  | QC3: 226 | 223 ± 5.29 | 98.7 | 2.37 |
|  |  | Cycle V | QC1: 3.19 | 2.87 ± 0.12 | 90.1 | 4.18 |
|  |  |  | QC3: 226 | 221 ± 15.0 | 97.9 | 6.78 |
|  | Ambient Temp. (22 °C) | 24 hrs. | QC1: 3.19 | 3.12 ± 0.05 | 97.9 | 1.64 |
|  |  |  | QC3: 226 | 206 ± 2.52 | 91.3 | 1.22 |
|  |  | 48 hrs. | QC1: 3.19 | 3.02 ± 0.11 | 94.8 | 3.54 |
|  |  |  | QC3: 226 | 203 ± 3.51 | 90.0 | 1.73 |
|  | Refrigerator (4 °C) | 24 hrs. | QC1: 3.19 | 3.16 ± 0.10 | 99.0 | 3.01 |
|  |  |  | QC3: 226 | 212 ± 2.65 | 93.8 | 1.25 |
|  |  | 48 hrs. | QC1: 3.19 | 3.23 ± 0.06 | 101 | 1.86 |
|  |  |  | QC3: 226 | 217 ± 4.00 | 96.0 | 1.84 |
|  | Bench Top (22 °C) | 4 hrs. | QC1: 3.19 | 3.04 ± 0.06 | 95.4 | 1.90 |
|  |  |  | QC3: 226 | 234 ± 8.50 | 104 | 3.63 |
|  | Extracted (stored at 4 °C) | 24 hrs | QC1: 3.19 | 2.99 ± 0.11 | 93.8 | 3.68 |
|  |  |  | QC3: 226 | 208 ± 2.08 | 92.2 | 1.00 |
|  | Auto sampler (4 °C) | 74 hrs. | QC1: 3.19 | 3.01 ± 0.16 | 94.2 | 5.16 |
|  |  |  | QC3: 226 | 236 ± 10.1 | 104 | 4.27 |
|  | Evaporated (stored at 4 °C) | 48 hrs. | QC1: 3.19 | 2.98 ± 0.10 | 93.4 | 3.41 |
|  |  |  | QC3: 226 | 228 ± 2.65 | 101 | 1.16 |
|  | Evaporated (stored at -80 °C) | 120 hrs. | QC1: 3.19 | 3.06 ± 0.19 | 95.8 | 6.29 |
|  |  |  | QC3: 226 | 261 ± 26.5 | 115 | 10.2 |
|  | Long Term (-80 °C) * | 1.6 years | QC1: 3.19 | 3.36 ± 0.10 | 105 | 2.90 |
|  |  |  | QC3: 226 | 245 ± 5.52 | 108 | 2.25 |
| Desethylamodiaquine | Freeze/ Thaw | Cycle I | QC1: 4.64 | 4.56 ± 0.17 | 98.2 | 3.74 |
|  |  |  | QC3: 524 | 512 ± 4.04 | 97.6 | 0.79 |
|  |  | Cycle III | QC1: 4.64 | 4.33 ± 0.04 | 93.4 | 0.96 |
|  |  |  | QC3: 524 | 498 ± 29.2 | 95.1 | 5.86 |
|  |  | Cycle V | QC1: 4.64 | 4.46 ± 0.11 | 96.0 | 2.55 |
|  |  |  | QC3: 524 | 526 ± 36.1 | 100 | 6.85 |
|  | Ambient Temp. (22 °C) | 24 hrs. | QC1: 4.64 | 4.49 ± 0.23 | 96.8 | 5.14 |
|  |  |  | QC3: 524 | 510 ± 16.1 | 97.3 | 3.15 |
|  |  | 48 hrs. | QC1: 4.64 | 4.34 ± 0.04 | 93.6 | 0.93 |
|  |  |  | QC3: 524 | 453 ± 5.00 | 86.5 | 1.10 |
|  | Refrigerator (4 °C) | 24 hrs. | QC1: 4.64 | 4.47 ± 0.22 | 96.4 | 4.86 |
|  |  |  | QC3: 524 | 460 ± 45.7 | 87.8 | 9.92 |
|  |  | 48 hrs. | QC1: 4.64 | 4.81 ± 0.15 | 104 | 3.15 |
|  |  |  | QC3: 524 | 487 ± 18.0 | 92.9 | 3.7 |
|  | Bench Top (22 °C) | 4 hrs. | QC1: 4.64 | 4.58 ± 0.12 | 98.7 | 2.52 |
|  |  |  | QC3: 524 | 478 ± 1.15 | 91.3 | 0.24 |
|  | Extracted (stored at 4 °C) | 24 hrs | QC1: 4.64 | 4.61 ± 0.24 | 99.4 | 5.14 |
|  |  |  | QC3: 524 | 509 ± 14.6 | 97.2 | 2.86 |
|  | Auto sampler (4 °C) | 74 hrs. | QC1: 4.64 | 4.71 ± 0.35 | 102 | 7.45 |
|  |  |  | QC3: 524 | 495 ± 13.8 | 94.5 | 2.80 |
|  | Evaporated (stored at 4 °C) | 48 hrs. | QC1: 4.64 | 4.59 ± 0.11 | 99.0 | 2.41 |
|  |  |  | QC3: 524 | 494 ± 10.3 | 94.3 | 2.08 |
|  | Evaporated (stored at -80 °C) | 120 hrs. | QC1: 4.64 | 4.74 ± 0.33 | 102 | 6.99 |
|  |  |  | QC3: 524 | 531 ± 5.29 | 101 | 1.00 |
|  | Long Term (-80 °C) * | 1.6 years | QC1: 4.64 | 5.29 ± 0.06 | 114 | 1.05 |
|  |  |  | QC3: 524 | 537 ± 14.3 | 102 | 2.67 |

*Five replicates of each QC1 and QC3 level were quantified.
